# Plitidepsin has potent preclinical efficacy against SARS-CoV-2 by targeting the host protein eEF1A

**DOI:** 10.1126/science.abf4058

**Published:** 2021-01-25

**Authors:** Kris M. White, Romel Rosales, Soner Yildiz, Thomas Kehrer, Lisa Miorin, Elena Moreno, Sonia Jangra, Melissa B. Uccellini, Raveen Rathnasinghe, Lynda Coughlan, Carles Martinez-Romero, Jyoti Batra, Ajda Rojc, Mehdi Bouhaddou, Jacqueline M. Fabius, Kirsten Obernier, Marion Dejosez, María José Guillén, Alejandro Losada, Pablo Avilés, Michael Schotsaert, Thomas Zwaka, Marco Vignuzzi, Kevan M. Shokat, Nevan J. Krogan, Adolfo García-Sastre

**Affiliations:** 1 Department of Microbiology, Icahn School of Medicine at Mount Sinai, New York, NY, USA.; 2 Global Health Emerging Pathogens Institute, Icahn School of Medicine at Mount Sinai, New York, NY, USA.; 3 Department of Microbiology and Immunology and Center for Vaccine Development and Global Health (CVD), University of Maryland School of Medicine, Baltimore, MD, USA.; 4 Quantitative Biosciences Institute (QBI), University of California, San Francisco, CA 94158, USA.; 5 J. David Gladstone Institutes, San Francisco, CA 94158, USA.; 6 QBI Coronavirus Research Group (QCRG), San Francisco, CA 94158, USA.; 7 Department of Cellular and Molecular Pharmacology, University of California, San Francisco, CA 94158, USA.; 8 Huffington Foundation Center for Cell-Based Research in Parkinson’s Disease, Department of Cell, Developmental, and Regenerative Biology, Black Family Stem Cell Institute, Icahn School of Medicine at Mount Sinai, New York, NY, USA.; 9 Research and Development Department, PharmaMar, 28770 Colmenar Viejo, Madrid, Spain.; 10 Viral Populations and Pathogenesis Unit, CNRS UMR 3569, Institut Pasteur, 75724 Paris Cedex 15, France.; 11 Howard Hughes Medical Institute, University of California, San Francisco, CA 94143, USA.; 12 Department of Medicine, Division of Infectious Diseases, Icahn School of Medicine at Mount Sinai, New York, NY, USA.; 13 Tisch Cancer Institute, Icahn School of Medicine at Mount Sinai, New York, NY, USA.

## Abstract

Severe acute respiratory syndrome coronavirus 2 (SARS-CoV-2) viral proteins interact with the eukaryotic translation machinery, and inhibitors of translation have potent antiviral effects. We found that the drug plitidepsin (aplidin), which has limited clinical approval, possesses antiviral activity (90% inhibitory concentration = 0.88 nM) that is more potent than remdesivir against SARS-CoV-2 in vitro by a factor of 27.5, with limited toxicity in cell culture. Through the use of a drug-resistant mutant, we show that the antiviral activity of plitidepsin against SARS-CoV-2 is mediated through inhibition of the known target eEF1A (eukaryotic translation elongation factor 1A). We demonstrate the in vivo efficacy of plitidepsin treatment in two mouse models of SARS-CoV-2 infection with a reduction of viral replication in the lungs by two orders of magnitude using prophylactic treatment. Our results indicate that plitidepsin is a promising therapeutic candidate for COVID-19.

## Hurting the virus by targeting the host

Many host proteins play a role in the life cycle of severe acute respiratory syndrome coronavirus 2 (SARS-CoV-2), and some are required for viral replication and translation. There are efforts toward finding drugs that target viral proteins, but a complementary approach is to target these required host proteins. White *et al.* explored the antiviral activity of the cyclic depsipeptide drug plitidepsin, which targets the hosts cell's translational machinery (see the Perspective by Wong and Damania). The authors show that in cells, the drug is substantially more potent than remdesivir against SARS-CoV-2, with limited cellular toxicity. Prophylactic treatment protected mice against SARS-CoV-2 infection, so further investigation of plitidepsin as a therapeutic is warranted.

*Science*, this issue p. 926; see also p. 884

## Teaser

Plitidepsin is a host-targeted antiviral against SARS-CoV-2, with potent efficacy both in vitro and in mouse models.

Over the past 20 years, three novel coronaviruses have been introduced into the human population, causing substantial morbidity and mortality. The severe acute respiratory syndrome coronavirus (SARS-CoV) and Middle East respiratory syndrome coronavirus (MERS-CoV) epidemics were each limited in scope, but both are associated with severe disease and high mortality rates (*1*–*3*). The ongoing COVID-19 pandemic caused by the SARS-CoV-2 virus is the result of a zoonotic transmission event, similar to previous coronavirus epidemics (*4*–*7*). Recent studies have detected many SARS-like and MERS-like coronaviruses in natural bat reservoirs and have shown them to be capable of replication in human lung cells in vitro (*8*–*10*). This suggests the presence of a large reservoir of coronaviruses with pandemic potential. Antiviral therapeutics are urgently needed to combat SARS-CoV-2 in the current pandemic and will be the first line of defense for the future coronavirus epidemics that appear more likely as the human population expands in close contact with animal reservoirs.

COVID-19 is a viral-induced inflammatory disease of the airways and lungs with multi-organ involvement that can cause severe respiratory and systemic issues. SARS-CoV-2 replication in the lungs leads to inflammatory, innate, and adaptive immune responses that cause substantial host tissue damage (*3*, *11*). COVID-19 can lead to end-stage lung disease and systemic involvement with currently limited treatment options and poor prognoses. The current standards of care include oxygen therapy and ventilation, along with the antiviral remdesivir and the anti-inflammatory dexamethasone. Remdesivir (*12*, *13*) and dexamethasone (*14*) have each improved patient outcomes in clinical trials and have been approved for emergency use by regulatory agencies, but remdesivir in particular has shown limited efficacy (*15*) and dexamethasone is a steroid that does not directly inhibit viral replication. This leaves a continued need for the development or repurposing of antiviral drugs for the treatment of COVID-19.

Our previously published SARS-CoV-2 (*16*) and pan-coronaviral (*17*) interactomes highlighted 332 host proteins that are likely to play a role in the viral life cycle of SARS-CoV-2. In that work we tested 47 existing drugs that were known to modulate these identified host proteins, with many of these drugs showing substantial antiviral activity against SARS-CoV-2 in cell culture (*16*). Of the inhibitors tested, those that targeted the eukaryotic translation machinery (eIF4H interacts with SARS-CoV-2 Nsp9) demonstrated particularly potent antiviral activities. Zotatafin (*18*), an inhibitor of eIF4A (a partner of eIF4H), had a 90% inhibitory concentration (IC_90_) of 154 nM, and ternatin-4 (*19*), an inhibitor of eEF1A that has potential interactions with multiple coronavirus proteins (*17*), had an IC_90_ of 15 nM against SARS-CoV-2 in Vero E6 cells (*16*).

## Plitidepsin is a potent inhibitor of SARS-CoV-2 in vitro

**Fig. 1:**
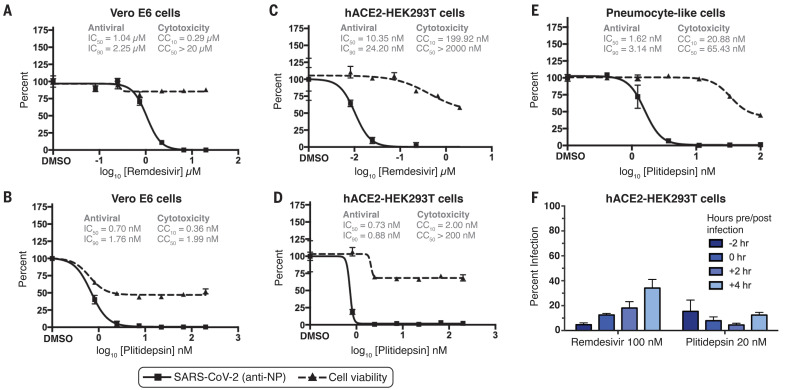
Plitidepsin exhibits a strong antiviral activity in SARS-CoV-2 multiple cell lines. (**A** to **E**) Vero E6 cells [(A) and (B)], hACE2-293T cells [(C) and (D)], or pneumocyte-like cells (E) were treated with indicated doses of remdesivir [(A) and (C)] or plitidepsin [(B), (D), and (E)]. IC_50_, IC_90_, 50% cytotoxic concentration (CC_50_), and CC_10_ values are indicated above the curves. All cells were pretreated for 2 hours and the drugs were maintained in the media throughout the experiment. SARS-CoV-2 infection and cell viability were measured at 48 hours. (**F**) The antiviral activities of plitidepsin and remdesivir were evaluated in pretreatment and post-infection time points in hACE2-293T cells. In all panels, data are means ± SD of three independent experiments performed in biological triplicate. DMSO, dimethyl sulfoxide.

In an effort to further explore the therapeutic potential of host eEF1A as a target for the treatment of COVID-19, we evaluated the eEF1A inhibitor plitidepsin (aplidin), which has limited clinical approval for the treatment of multiple myeloma. Plitidepsin has also successfully undergone a phase I/II clinical study for the treatment of COVID-19 (*20*, *21*) by the pharmaceutical company PharmaMar and is moving forward into a phase II/III COVID-19 study. We first tested plitidepsin inhibition of SARS-CoV-2 replication using an immunofluorescence-based antiviral screening assay in Vero E6 cells (*22*). Plitidepsin inhibited SARS-CoV-2 with an IC_90_ of 1.76 nM (Fig. 1B), which was 9 times as potent as ternatin-4 and 87.5 times as potent as zotatafin in the same assay (*16*). We next tested plitidepsin in the same antiviral assay using a human cell line (hACE2-293T). The anti–SARS-CoV-2 activity of plitidepsin was even more potent in human cells, with an IC_90_ of 0.88 nM (Fig. 1D), which is more potent than remdesivir tested in the same cell line by a factor of 27.5 (Fig. 1C). The cytotoxicity of plitidepsin was examined in parallel with antiviral activity; at all concentrations in both cell types, we observed a cytostatic impact on cell proliferation (Fig. 1, B and D). We had previously found the lack of a dose-response curve in our cytotoxic assay to be suggestive of a cytostatic, rather than cytotoxic, effect on cells, but further work is required to confirm this hypothesis. Finally, we tested the antiviral effect of plitidepsin in an established model of human pneumocyte-like cells (*23*, *24*). We found that treatment with plitidepsin inhibited SARS-CoV-2 replication (Fig. 1E) with an IC_90_ of 3.14 nM and a selectivity index of 40.4, which suggests that plitidepsin has potent antiviral activity in primary human lung cells.

In an effort to better understand the mechanism of action through which plitidepsin inhibits SARS-CoV-2 infection, we performed a time-of-addition assay in which plitidepsin or remdesivir was added to hACE2-293T cells at –2, 0, +2, or +4 hours relative to infection. In this 8-hour single-cycle infection, we found that 20 nM plitidepsin strongly inhibited nucleocapsid protein expression even when added 4 hours after infection (Fig. 1F). This is indicative of a cytoplasmic replication-stage inhibitor, which is consistent with the predicted antiviral mechanism of a known translation inhibitor.

Remdesivir is part of the current standard of care for the treatment of COVID-19 (*25*, *26*). We therefore assessed the dynamics between the antiviral effects of plitidepsin and remdesivir when used together in vitro. Our analysis using the Synergyfinder (*27*) software suggests that plitidepsin has an additive effect with remdesivir (fig. S2) and would be a potential candidate to be considered in a combined therapy with the current standard of care.

## Plitidepsin antiviral activity against SARS-CoV-2 is mediated through the inhibition of eEF1A

**Fig. 2:**
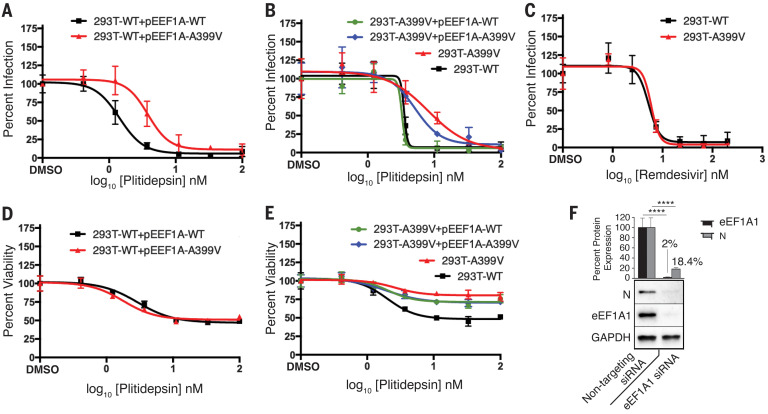
Antiviral mechanism of action of plitidepsin is mediated through inhibition of eEF1A. (**A**) Plitidepsin inhibition of SARS-CoV-2 replication in 293T cells transfected with eEF1A-WT or eEF1A-A399V expression vectors. Plitidepsin inhibition is reduced by expression of the A399V mutation, whereas virus replication in wild-type and eEF2A-transfected mutations remain susceptible to treatment with plitidepsin. (**B** and **C**) Plitidepsin (B) and remdesivir (C) inhibition of SARS-CoV-2 replication in a CRISPR 293T cell line carrying an A399V mutation in eEF1A. Viral replication in wild-type eEF1A preserves susceptibility to plitidepsin inhibition, whereas the presence of the eEF1A A399V mutation rendered the SARS-CoV-2 infection resistant to the eEF1A inhibitor. Remdesivir inhibition of SARS-CoV-2 viral replication was not affected by the A399V mutation. (**D** and **E**) Plitidepsin inhibition of cell proliferation, as measured by (4,5-dimethylthiazol-2-yl)-2,5-diphenyltetrazolium bromide (MTT) assay, is not affected by transfection of the A399V mutant (D) but is reduced by the 293T-A399V CRISPR cell line (E). (**F**) siRNA silencing of eEF1A greatly reduces N protein levels. In all panels, data are means ± SD of three independent experiments performed in biological triplicate. ****P < 0.0001.

Plitidepsin inhibits the activity of the host factor eEF1A and is predicted to interact with the same binding site as didemnin B, which is structurally related to plitidepsin, and the structurally unrelated ternatin-4. Exogenous overexpression of an Ala^399^ → Val (A399V) mutant of eEF1A confers resistance in cancer cells to both didemnin B (*28*) and ternatin-4 (*29*) inhibition, and we predicted that it may similarly affect plitidepsin. We examined whether this A399V mutation could mitigate the observed anti–SARS-CoV-2 activity of plitidepsin. First, we transiently cotransfected 293T cells with expression plasmids for hACE2 and either wild-type eEF1A (eEF1A-WT) or eEF1A-A399V, which were confirmed to be expressed in ~30% of cells by means of immunofluorescent staining for the Flag epitope (fig. S1). We then measured the antiviral activity of plitidepsin against SARS-CoV-2 in these transfected 293T cells. Transfection with eEF1A-A399V, but not eEF1A-WT, increased the IC_90_ of plitidepsin by a factor of >10 (Fig. 2A). No impact from the A399V mutant transfection was observed upon plitidepsin inhibition of cell proliferation (Fig. 2D), consistent with observations of ternatin-4 (*29*). The differential effect of eEF1A-A399V transient transfection between the antiviral and antiproliferative impact of plitidepsin is consistent with previous findings that coronaviruses are considerably more sensitive to translation perturbations than the host cell (*30*, *31*). We then generated an eEF1A-A399V CRISPR knock-in 293T cell line (293T-A399V) to further evaluate the role of eEF1A inhibition in the antiviral activity of plitidepsin. We found that this 293T-A399V cell line was refractory to the SARS-CoV-2 antiviral activity of plitidepsin by a factor of >12 as compared to the parental cell line (Fig. 2B) but did not have a similar impact on remdesivir inhibition (Fig. 2C). Furthermore, we found that plitidepsin antiviral activity could be almost fully restored through transient transfection of the 293T-A399V cells with wild-type, but not mutant, eEF1A (Fig. 2B). This 293T-A399V cell line was also resistant to the antiproliferative activity of plitidepsin, and this could only be partially rescued by transfection of the wild-type protein (Fig. 2E), again similar to previous results with ternatin-4 (*29*). Furthermore, small interfering RNA (siRNA) silencing of eEF1A protein expression during SARS-CoV-2 infection led to a large reduction in viral N protein levels but had no impact on the GAPDH control (Fig. 2F). Taken together, this evidence indicates that the antiviral activity of plitidepsin is mediated through eEF1A inhibition and confirms eEF1A as a druggable target for the inhibition of SARS-CoV-2 replication.

**Fig. 3:**
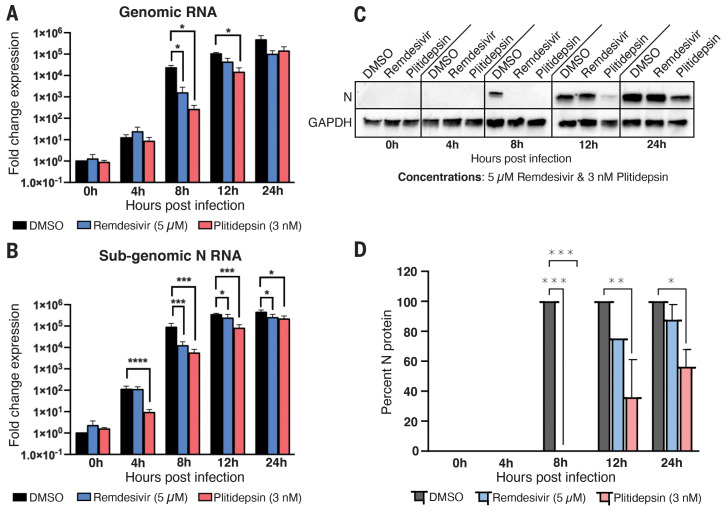
Plitidepsin treatment causes a specific reduction in subgenomic RNA expression. (**A** to **D**) Vero E6 cells were infected with SARS-CoV-2 at an MOI of 1 in the presence or absence of 3 nM plitidepsin or 5 μM remdesivir and samples were taken at the indicated time points. The levels of genomic RNA (A) and subgenomic N RNA (B) were analyzed with specific reverse transcription quantitative polymerase chain reactions (RT-qPCR). (C) Cell lysates were collected at the indicated times and subjected to Western blotting. (D) Each protein band was quantified by ImageJ and normalized to GAPDH levels. Data are means ± SD of three independent experiments performed in biological triplicate. *P < 0.05, **P < 0.01, ***P < 0.001, ****P < 0.0001.

We next explored the impact of plitidepsin treatment on viral RNA and protein production over the course of SARS-CoV-2 infection. We analyzed the SARS-CoV-2 genomic and N subgenomic RNA content of Vero E6 cells infected with SARS-CoV-2 at a multiplicity of infection (MOI) of 1 at 4, 8, 12, and 24 hours after infection in the presence or absence of equivalent inhibitory doses of plitidepsin or remdesivir. We found that plitidepsin significantly reduced genomic RNA content at 8 and 12 hours after infection and fell just short of significance at the 24-hour time point, similar to remdesivir treatment (Fig. 3A). Interestingly, plitidepsin had a much greater impact on the accumulation of the N subgenomic RNA. Plitidepsin greatly reduced the subgenomic RNA expression as early as 4 hours after infection and maintained a significant impact throughout the time course (Fig. 3B). Remdesivir had no effect on N subgenomic RNA at 4 hours, but did show a reduction at all other time points tested. We then measured the viral N protein levels in the presence and absence of plitidepsin or remdesivir treatment. Similar to RNA levels, plitidepsin had a more potent and sustained inhibition of the expression the N protein over the time course of infection relative to remdesivir (Fig. 3, C and D). This specific inhibition of N subgenomic RNA expression, particularly early in infection, is likely a result of the inhibition of viral translation by plitidepsin. It was previously shown that coronaviruses are highly sensitive to translation inhibitors (*30*, *31*) and that negative-sense genome accumulation is more greatly affected than the positive sense (*32*). The current model of coronavirus discontinuous transcription (*33*) has been guided by evidence that subgenomic RNA formation occurs during negative strand synthesis (*34*). Therefore, a translation inhibitor that has a greater impact on negative-sense RNA production would also be expected to specifically reduce subgenomic RNA formation and accumulation, as we observed with plitidepsin. Furthermore, consistent with an impact of plitidepsin in protein translation, N protein levels were more greatly reduced in plitidepsin-treated cells than in remdesivir-treated cells at 24 hours after infection, when levels of N RNA were equivalent between these two treatments.

## Plitidepsin shows in vivo antiviral efficacy in mouse models of SARS-CoV-2 infection

Plitidepsin has been clinically developed for the treatment of multiple myeloma with a well-established safety profile and pharmacokinetics (*35*–*38*). Initially, plitidepsin underwent a large clinical development program in which cancer patients were treated with plitidepsin as a single agent in several phase I and II clinical trials. Results gathered from these clinical studies demonstrated that the probability of having cardiac adverse events, a concern in COVID-19 patients, was not significantly affected by plitidepsin treatment (*39*–*41*), although these events were found in other chemically related compounds that display a different mechanism of action (*42*, *43*). It is worth highlighting that plitidepsin had a good safety profile in a phase I clinical trial (*44*), which administered a total of 11.4 mg spaced over 5 days of treatment. The dose level used in the COVID-19 proof-of-concept phase I study (*21*) had a maximum total of 7.5 mg spaced over 3 days.

**Fig. 4:**
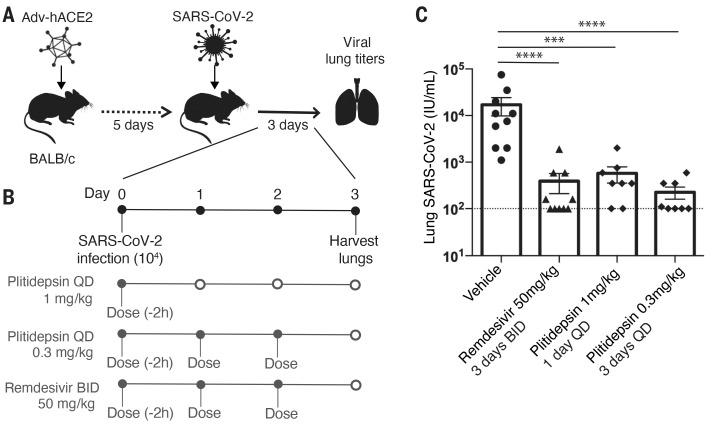
Plitidepsin treatment significantly reduces SARS-CoV-2 infection in BALB/c mice expressing human ACE2. (**A**) Schematic of adenovirus expression of human ACE2 model of SARS-CoV-2 infection. BALB/c mice were transduced with human ACE2 expressing adenovirus. Mice were sensitized intranasally with 2.5 × 10^8^ pfu. (**B**) Mice were intranasally infected with 10^4^ pfu of SARS-CoV-2 and subcutaneously treated with either 0.3 mg/kg plitidepsin once daily for 3 days, a single dose of 1 mg/kg plitidepsin, or 50 mg/kg remdesivir once daily for 3 days. (**C**) SARS-CoV-2 lung titers in the plitidepsin-treated group relative to vehicle and remdesivir controls. Virus titers were determined in whole lung homogenates by median tissue culture infectious dose (TCID_50_) at day 3 after infection. The limit of detection for viral titers is indicated with a dotted line. Vehicle and remdesivir, *N* = 10; plitidepsin 1 mg/kg and 0.3 mg/kg, *N* = 8. ***P < 0.001, ****P < 0.0001.

On the basis of these clinical safety data and good pharmacokinetics (fig. S3A), we determined that a concentration of plitidepsin an order of magnitude greater than the demonstrated in vitro IC_90_ could be safely achieved in the lungs of mice using a single daily dose. Therefore, we tested the in vivo efficacy of plitidepsin in two different established animal models of SARS-CoV-2 infection. First, we used a human ACE2-expressing adenovirus to transduce the naturally resistant wild-type BALB/c mice and sensitize them to SARS-CoV-2 infection (Fig. 4A) (*45*). Five days after adenovirus transduction, mice were infected with 1 × 10^4^ plaque-forming units (pfu) of SARS-CoV-2. As a proof-of-principle experiment, we performed prophylactic dosing with 0.3 mg/kg or 1 mg/kg plitidepsin 2 hours before infection with SARS-CoV-2. The 0.3 mg/kg group received continued dosing once per day for 2 more days, whereas the 1 mg/kg group received only that single dose (Fig. 4B). SARS-CoV-2 lung titers were quantified from two independent experiments for the plitidepsin groups and compared to vehicle and remdesivir controls (Fig. 4C). There was a reduction of nearly 2 log units in SARS-CoV-2 viral titers in the lungs of the 0.3 mg/kg plitidepsin group relative to the vehicle control group, whereas there was a reduction of 1.5 log units observed from the single dose of 1 mg/kg plitidepsin. Note that we used a very high concentration of remdesivir in these assays (50 mg/kg) because of the known high concentration of esterases present in mouse serum that degrade remdesivir (*46*).

**Fig. 5:**
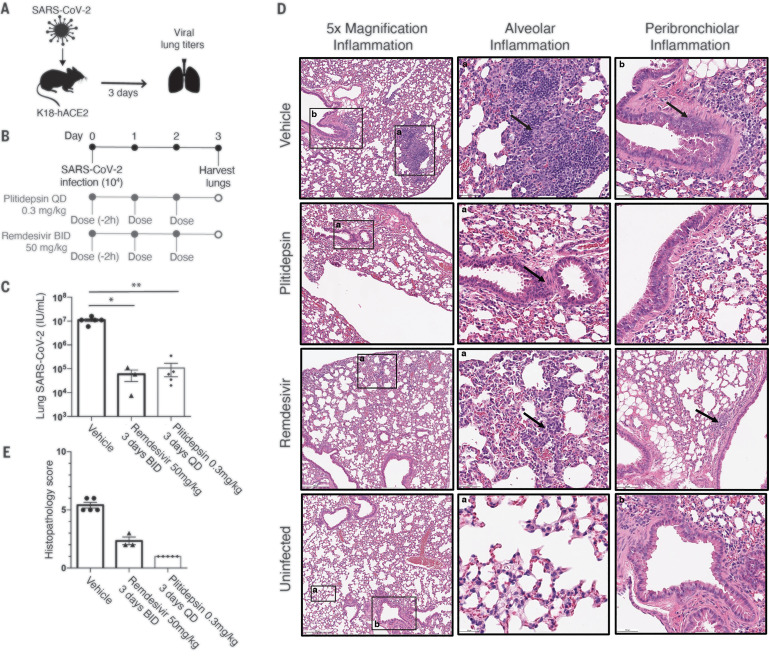
Plitidepsin shows in vivo antiviral efficacy in the K18-hACE2 mouse model. (**A**) Schematic of the K18-hACE2 model of SARS-CoV-2 infection. (**B**) Mice were intranasally infected with 10^4^ pfu of SARS-CoV-2 and subcutaneously treated with 0.3 mg/kg plitidepsin once daily for 3 days or with 50 mg/kg remdesivir twice daily for 3 days. (**C**) SARS-CoV-2 lung titers in the plitidepsin-treated group relative to vehicle and remdesivir controls. Virus titers were determined in whole lung homogenates by TCID50 at day 3 after infection. Five mice were used in each group, except for the remdesivir control, which had 3. *P < 0.05, **P < 0.01. (**D**) Lungs were harvested on day 3 after infection, paraffin-embedded, and 5-μm sections stained for hematoxylin and eosin. Regions of the lung anatomy where inflammation was assessed are highlighted by black boxes, with the corresponding higher-magnification image indicated by matching letter. Regions where inflammation was detected are indicated by arrows. (**E**) Pathological severity scores in infected mice. To evaluate comprehensive histological changes, lung tissue sections were scored according to pathological changes outlined in the supplementary materials.

We then performed a study in the K18-hACE2 mouse model (Fig. 5A), which supports a robust SARS-CoV-2 infection (*45*, *47*), in which the 0.3 mg/kg dosage of plitidepsin was assessed for ability to reduce viral titers and inflammation in the lung. K18-hACE2 mice were treated with one daily dose of plitidepsin for 3 days starting 2 hours before infection with SARS-CoV-2 (Fig. 5B). We found a reduction of 2 log units in viral lung titers at day 3, similar to two daily 50 mg/kg doses of remdesivir (Fig. 5C). Histopathology analysis (Fig. 5D) also showed a reduction of lung inflammation in plitidepsin-treated mice (histopathology score of 1/16) over vehicle-treated (histopathology score of 5.4/16) and remdesivir-treated (histopathology score of 2.3/16) mice at day 3 after infection (Fig. 5E). There was no peribronchiolar inflammation noted in the plitidepsin-treated group. Taken together, these experiments show that plitidepsin treatment can reduce the replication of SARS-CoV-2 by two orders of magnitude and reduce lung inflammation in vivo, and has compelling potential for clinical efficacy for the treatment of COVID-19.

## Discussion

The ongoing SARS-CoV-2 pandemic has created the immediate need for antiviral therapeutics that can be moved into the clinic within months rather than years. This led us to screen clinically approved drugs with established bioavailability, pharmacokinetics, and safety profiles. Our previous study of the SARS-CoV-2 interactome (*16*) led us to eEF1A as a druggable target with the potential for potent inhibition of SARS-CoV-2 in vitro. eEF1A has been previously described to be an important host factor for the replication of many viral pathogens (*48*–*50*), including influenza virus (*51*) and respiratory syncytial virus (*52*). Specifically, it has been found to be involved in transmissible gastroenteritis coronavirus replication (*53*) and was detected in SARS-CoV virions (*54*). Therefore, inhibition of eEF1A as a strategy for the treatment of viral infection may extend to other human coronaviruses and beyond to unrelated viral pathogens. This potential for broad-spectrum antiviral activity makes plitidepsin an intriguing candidate for further exploration as a treatment for viral infections with no clinically approved therapeutics. It is also important to note that a host-targeted antiviral such as plitidepsin offers protection from naturally occurring viral mutants, to which viral-targeted therapeutics and vaccines are more susceptible. In fact, plitidepsin was found to maintain nanomolar potency against the B.1.1.7 variant (*55*) recently discovered in the United Kingdom (*56*, *57*).

In our animal experiments, we did detect a slight body weight loss of mice that were treated with plitidepsin daily, whereas mice that received a single 1 mg/kg dose did not lose any weight while still exhibiting reductions in viral lung titers (fig. S3B). It is unclear whether this observed toxicity is mouse-specific, and although toxicity is a concern with any host-targeted antiviral, the safety profile of plitidepsin is well established in humans. Furthermore, the dose of plitidepsin being used in an ongoing COVID-19 clinical trial is substantially lower than used in these experiments and it has been well tolerated in patients with minimal side effects. Interestingly, the most well-established and effective steroid for the treatment of COVID-19, dexamethasone (*14*), is also a commonly used treatment for multiple myeloma (*58*). This has led to plitidepsin already having an established safety profile with concurrent dexamethasone treatment (*59*, *60*) and should allow for clinicians to treat with both drugs if warranted. This study establishes plitidepsin as a host-targeted anti–SARS-CoV-2 agent with in vivo efficacy. Our data and the initial positive results from PharmaMar’s clinical trial suggest that plitidepsin should be strongly considered for expanded clinical trials for the treatment of COVID-19.

## Supplementary Materials

science.sciencemag.org/content/371/6532/926/suppl/DC1 Materials and Methods Figs. S1 to S3 References (*61*–*64*) MDAR Reproducibility Checklist
